# A Facile Technique to Extract the Cross-Sectional Structure of Brittle Porous Chars from Intumescent Coatings

**DOI:** 10.3390/polym11040640

**Published:** 2019-04-09

**Authors:** Gizem Okyay, Fabienne Samyn, Maude Jimenez, Serge Bourbigot

**Affiliations:** Univ. Lille, CNRS, INRA, ENSCL, UMR 8207 - UMET - Unité Matériaux et Transformations, F-59000 Lille, France; gizem.okyay@gmail.com (G.O.); fabienne.samyn@univ-lille.fr (F.S.); maude.jimenez@univ-lille.fr (M.J.)

**Keywords:** intumescent coating, porous char, fractals, digital microscopy, color contrasting, image retrieval

## Abstract

Intumescent coatings are part of passive fire protection systems. In case of fire, they expand under thermal stimuli and reduce heat transfer rates. Their expansion mechanisms are more or less recognized, but the fire testing data shall be interpreted as function of coating morphology. Expansion ratios are examined together with the inner structures of specimens submitted to fire. Bare cutting techniques damage the highly porous and fibrous specimens because they become very crumbly due to charring. So far, absorption contrasted X-ray computed microtomography (CT) was used as a non-destructive technique. Nevertheless, access to X-ray platforms can be relatively expensive and scarce for regular use. Also, it has some drawbacks for carbon rich specimens strongly adhering on steel substrates because it leads sometimes to noisy images and lost data due to resolution limits on specimens reaching ten of centimeters. Therefore, we propose an inexpensive and more accessible experimental approach to observe those specimens with minimized structural damage under visible lighting. To that end, charred specimens were casted into pigmented epoxy resin. After surface treatments, color contrasted cross-sections could be observed under optical digital microscopy thanks to high level of interconnectivity of pores. Subsequent image treatments confirmed that the structural integrity was kept when compared to previous CT data. The proposed method is practical, cheaper and more accessible for the quantitative assessment of inner structure of charred brittle specimens.

## 1. Introduction

### 1.1. Context and Motivation

Fire protective coatings are monitored in situ or ex situ according to various fire scenarios, in order to understand material reaction and resistance to fire. Intumescent paintings [1], a subset of those coatings, can expand up to 500% of their initial thickness when exposed to fire. Their porous morphology evolves as a function of external stimuli; and alongside their chemical composition and expansion ratio, their macro and microstructures modify their performance criteria for thermal protection and structural resistance [2].

In literature, the cross-sections of intumescent chars were reconstructed by X-ray computed tomography (CT) [3,4] or the interior structure of specimens were imaged by direct cutting at ambient conditions [4,5]. Size of specimens being in the macroscale (reaching a few centimeters), their transparency is only possible under X-rays for a non-destructive 3D volume reconstruction. For example, Morys et al. [4] examined the material density, cell and strut orientations using CT, indicating the importance of internal patterns not limited to expansion ratio nor to apparent porosity. They observed only qualitatively the microscale (under optical or electron microscopy) by bare cutting of specimens. Muller et al. [3] studied simultaneously the X-ray CT data with thermal conductivity, observing qualitatively chemical elemental distribution throughout expansion. Staggs [6] examined the thermal conductivity as a function of fitted porosity to CT geometry and also performed physics simulations upon geometries obtained by photography/photocopy of bare cut cross-sections. Further examples on the use of morphology for fire protection can be found in the works of Cirpici et al. [7] and references therein. On the other hand, very recently, Okyay et al. [8] demonstrated the existence of power laws in expansion and charring of intumescent coatings: the evolution of micro and macrostructure could be defined by fractal rules and generalized for different intumescent actions (i.e., physical or chemical [9]), as complementary to phenomenological approaches [10]. For such studies, measurable accurate cross-sectional images were compulsory using a combination of CT and correlative imaging, in order to describe the coating expansion patterns. Therefore, all the above research underlines the importance of accurate and precise quantifiable data of internal structure, to define and to characterize fire protective properties of expanded coatings.

### 1.2. Challenge and Scope of Study

Fire exposed specimens become very crumbly and brittle due to charring. It is difficult to keep their structural integrity while cutting the specimen through macropores and fibrous additives. So, direct cutting can be destructive and the consequent bare imaging (optical or electron microscopy on cut specimens) can fail to provide an accurate quantitative measurement of cross-sectional structure. The most used technique so far was the absorption contrasted X-ray CT. However, this has some drawbacks in specific cases. Indeed, significant scattering of rays can occur for coatings strongly adhering on steel substrates or for materials such as intumescent polymer metal laminates (multilayer materials alternating aluminium and intumescent coating layers [5]) and could be problematic and even prevent such measurements. Noise can be observed at HRCT (high resolution CT: either a local CT or acquisition on mm sized specimens) due to low absorption contrast of high carbon content. These are susceptible to lead to noisy reconstruction and lost data. Furthermore, the material density (porosity) and tortuosity obtained by CT is only apparent. The resolution of CT is limited by macroscale size of our specimens (reaching tenth of centimeters) as was mentioned by Morys et al. [4]. In between those macroscale specimens, reconstructions are limited to a resolution of around hundred of microns using X-ray sources available at bench-scale tomography: this is unable to catch micro porosity and tortuosity with individual additives. In this case, one needs homogenization and up/down-scaling for physical data. Otherwise, one shall cut the sample for microscopy or for local volume CT (i.e., HRCT), but the reconstruction noise is increased so the accuracy shall be reduced. Here, testing of smaller virgin coatings is not a feasible solution, because they shall not be representative of a real scale fire scenario. Yet, access to X-ray CT platforms can become rapidly time consuming, expensive and scarce, especially for high-resolution reconstructions or phase contrasted tomography. All the reasons above leaded us to investigate new techniques for the analysis of interior structure of coatings with minimized damage.

In this study, we propose a practical and cheaper solution to obtain the cross-sectional morphology of intumescent specimens using image retrieval. Fire tested and expanded coatings were casted into a pigmented epoxy resin. Cured specimens were cut and polished to obtain the cross-sections of interest, and then observed under optical digital microscopy.

## 2. Materials and Methods

### 2.1. Intumescent Coatings

**Figure 1:**
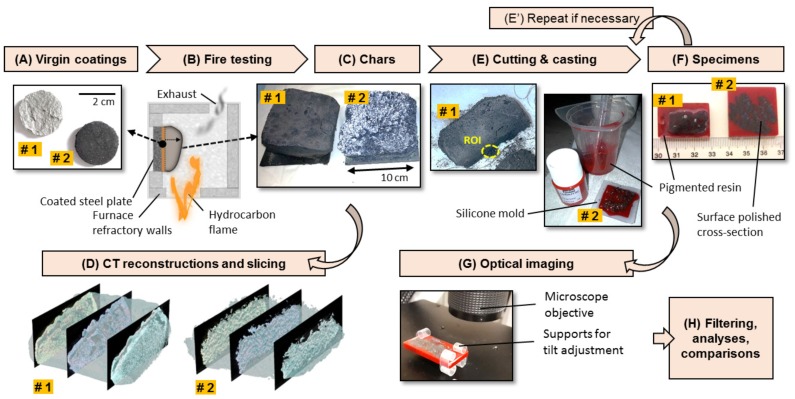
Overview of the workflow. Labels #1 and #2 indicate the specimen type, i.e., epoxy-based (for chemical expansion action) and silicone-based (for physical expansion action) coatings respectively. Images under their corresponding title: (**A**) Small specimens of virgin coatings. (**B**) Schematic illustration of fire testing facility [8]. (**C**) Charred specimens collected at the end of fire testing; they adhere to their underlying steel substrates. (**D**) X-ray CT 3D reconstructions of intact chars, adapted from Okyay et al. [8]. (**E**) Cutting (left) and resin casting (right). ROI is the region of interest. (**E’**) Resin casting can be repeated to fill the void sections on the cut surface, if the specimen was not fully impregnated due to isolated pores. (**F**) Surface polished specimens; numbered ticks of the ruler are on cm scale. (**G**) Digital microscopy on resin casted specimens.

Specimens were obtained from two different coatings as presented in Figure 1A. Coating #1 was a classical epoxy-based system designed for UL1709 fire testing standard, involving commercial formulation based on conventional recipe involving APP-based molecules, char former and blowing agent. Coating #2 was a silicone-based system, a partly in-house mixture containing silicone binder and expandable graphite for thermal expansion, designed for ISO834 and UL1709 fire scenarios. Samples for fire tests, catalysts, and primers were supplied by Dow Corning (DC, Seneffe, Belgium) and Advanced Insulations Systems (AIS, Gloucester, UK). Both systems were supplied in two parts as a base and a curing agent. Coating #2 contained EG350 in addition to silicone binder (Graphitwerk Kropfmuhl, Hauzenberg, Germany) [11]. Coating mixtures were prepared and applied on 10 × 10 × 0.3 cm^3^ steel plates at room temperature. Virgin coating thickness was 6 mm for epoxy-based and 4 mm for silicone-based systems. Steel plate preparations, degreasing, coating and curing were accomplished according to established protocols both for the epoxy and silicone-based mixtures respectively [11,12]. Steel plates (grade XC38 from Tartaix, Paris, France) were degreased with ethanol, sand blasted (Normfinish, Jean Brel SA, Stains, France) with aluminum oxide (particle size of 355–500 μm, Guyson, Chambly, France) at 5 bars pressure. Prior to Coating #2 application, primer was applied on steel plate to enhance the adhesion of the system in vertical configuration of fire exposure.

### 2.2. Fire Exposure

The dried/cured specimens were subjected to hydrocarbon fire in bench scale furnace following engineering standard [11,12] mimicking UL1709 normalized temperature versus time curve. The testing furnace was covered with refractory fiber panels stable up to 1300 °C, and the substrate was placed in front of propane burners leading to the heat induced expansion, as sketched in Figure 1B. The testing was stopped when the measured backside temperature of the steel plate reached 400 °C, the adopted standard value for normally loaded steel structural components. The coating specimens were entirely expanded and charred. Once the samples were cooled down, they were collected together with their steel plate as shown in Figure 1C. The expanded charred specimens obtained from epoxy-based and silicone-based coatings will be named thereby as Specimen #1 and Specimen #2 respectively.

### 2.3. Specimen Cutting

After cooling down of charred substrates, small cubes of approximately 2 cm^3^ were cut near the ROI (region of interest) of Specimens #1 and #2, as presented in Figure 1E, to keep the ROI intact. ROI is the middle bottom part presumed to be the active layer leading to continuous expansion during fire. Charred specimens were very crumbly, they needed to be cut with a very thin wire (here, dental floss was used). Specimen surfaces were covered gently with sticky material (here, fixing hairspray was used) to minimize the breaking into pieces during and after cutting.

### 2.4. Colored Resin Casting

Cut specimens were dried in an oven at 40 °C for 15 min to remove moisture. They were casted in silicone jewelery molds, at ambient temperature, into an epoxy resin mixed with pigments (Figure 1E,F). A low viscosity and high clarity epoxy resin of decoration (Castin′ Craft) was used. It ensured good impregnation into pores and distinct red color hue on optical images. Also, structural integrity of interior ROI was kept by resin casting.

Pigmentation was selected to have a color different than the natural color of char specimen (grayish-yellowish for Specimen #1, white-grayish for Specimen #2). Red pigments (Pebeo Ceramic) were incorporated into resin mixture for an opaque illusion. Reasons were twofold: creating good color contrast against the natural color of specimens, and minimizing penetration depth of light in order to prevent shadows behind the surface of interest. Using this technique, we were able to discriminate the cross-sectional structure of chars from the resin filled voids. Pigment size (around 50 μm) was rather adapted for our desired resolution limit because it was equal or smaller than the size of components/additives inside the charred coatings. Even though pigments of that size could not penetrate with the casting resin into smallest micropores (few tens of microns), they limited the propagation of light inside the specimen, allowing an improved brightness contrast for those micropores. This latter will be reminded in the results section during the discussion of color intensity histograms of optical recordings.

### 2.5. Surface Preparation

Resin casted specimens were cured at ambient temperature for at least 48 hours. After curing, too thick specimens were cut near the cross-section of interest by sawing. Surfaces were progressively trimmed down using silicon carbide abrasive papers (from P80 to P4000 grit, from ESCIL, Chassieu, France). The idea is to keep the surface roughness as small as possible (because the magnification was performed up to ×100 with 2.1 μm/pix), so, surfaces were polished with diamond pastes down to a micron (6, 3 and 1 μm, supplied from ESCIL, Chassieu, France).

### 2.6. Image Recordings

Polished surfaces represented the cross-sections of interest of charred specimens (Figure 1E). They were imaged using optical digital microscopy (model VHX-1000 from KEYENCE, Japan). Surface tilt was adjusted using flexible plastic clipses (from ESCIL, Chassieu, France), as shown in Figure 1G: focus was the same on the whole surface area up to ×200 magnification. Regular and HDR (high definition range) images were captured on relatively large surface areas (up to 2 cm^2^) by image stitching, at ×20, ×30, ×50 and ×100 magnifications using VHX-1000 microscopy suite. Subsequent image resolutions were 9.62 μm/pix (×20), 6.76 μm/pix (×30), 4.17 μm/pix (×50) and 2.12 μm/pix (×100). Please note that the HDR function captured multiple frames of an image in varying degrees of brightness by changing the shutter speed; microscopy suite composed them immediately and automatically into a single high-resolution image [13]. One can encounter glaring objects in intumescent chars such as expandable graphite flakes or mineral additives. So, capturing a broader brightness spectrum with HDR enabled greater image accuracy for those glaring objects [13]. Finally a full co-axial illumination, available by default for this optical microscope, was used in order to prevent artefacts that an uneven illumination could produce.

### 2.7. Image Treatments

**Figure 2:**
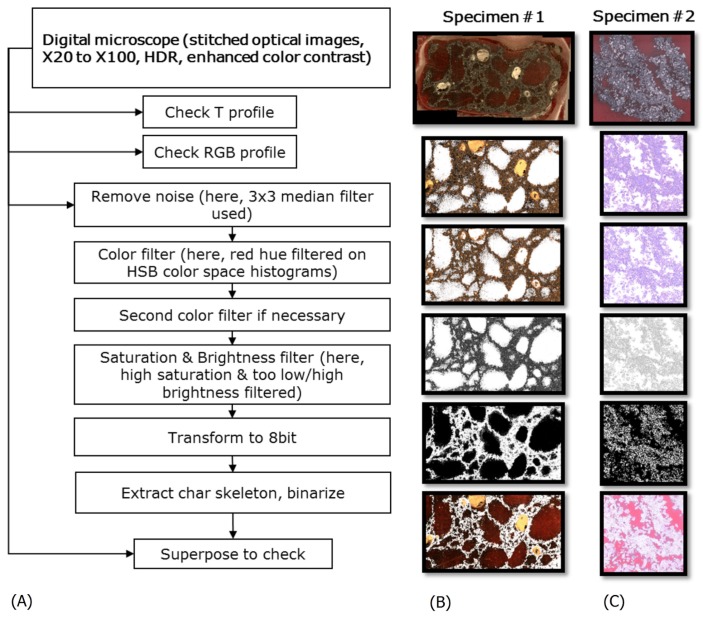
(**A**) Workflow for image retrieval of char skeleton; (**B**,**C**) Example images for sequential masking of the cross-sections of interest on polished surfaces #1 and #2 respectively.

Optical images were thresholded by color intensity profiling. Example surfaces were profiled to observe their texture (named hereby as T-profiles) using VHX-1000 microscopy suite. Corresponding RGB (red-green-blue) color profiles were extracted using Color Profiler plugin [14]. Once the optical behaviors of specimens were understood in the visible range, image extraction was performed in ImageJ [15] by sequential filtering in HSB (Hue, Saturation, Brightness) space [16]. HSB was the most suitable color system for surface imaging while providing the best histograms for the recognition of peak separations on those histograms [15,16]. Steps listed in Figure 2 will be discussed in Results.

### 2.8. Image Analyses

In order to check the accuracy of the optical method proposed, results were compared to X-ray CT data, formerly reported on same types of specimens [8]. To that end, fractal dimensions were computed upon power laws plotted using box counting technique [17] to define 2D solid skeleton of charred specimens. The analyses were repeated on images with variable magnifications (×20 to ×100) to check the self-similarity and to verify the lower limit of scale-invariant property in optical technique as compared to reported HRCT data [8].

## 3. Results and Discussion

Solid skeleton of chars were extracted by color contrasting. When the illumination was fairly even under optical microscopy, the method allowed an accurate enough definition of specimen morphology while keeping the structural integrity at maximum. This is verified by comparing the microscopy results with previous data obtained from CT. Therefore, we established a cheaper and more accessible metrology as depicted in Figure 1.

### 3.1. Understanding the Color Contrast on Resin Casted Specimens

**Figure 3:**
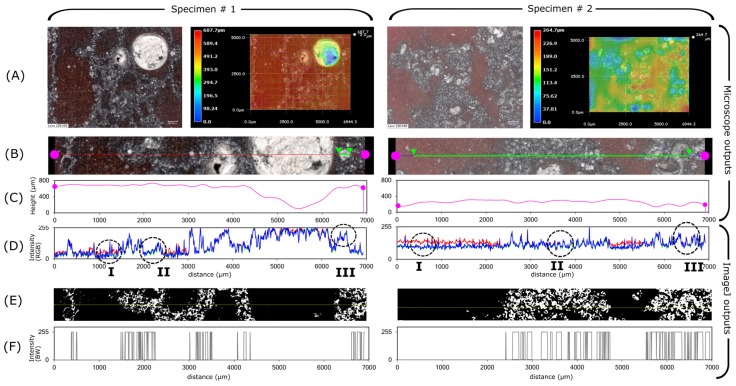
Preliminary observations by profile matching for specimens #1 and #2. (**A**) Original color contrasted optical images with their T-profile maps. (**B**) Selected lines for height and color profiles. (**C**) T-profiles (surface texture) corresponding to selected lines. (**D**) Corresponding intensity profiles in RGB space. (**E**) Preliminary binarization giving the porous char structure (solid in white, void in black). (**F**) Intensity profile of the binarized image through the selected lines.

Specimens collected at the end of fire testing were presented in Figure 1C. Specimen #1 is based on chemical intumescent action containing carbon and acid sources: the blowing agent ensures the bubbling reactions while the fiber additives ensure the structural integrity of char. The geometrical building blocks are the bubbly pore networks as seen in Figure 3A-left. Specimen #2 is based on a physical intumescent action containing intercalated expandable graphite (EG) as main additive, bound with silicone: the sublimation of intercalation compound (sulfate-based molecule) between graphite flakes ensures the expansion while the silicone binder ensures the structural integrity. The geometrical building blocks were formed of strata of EG/silicone, as seen in Figure 3A-right.

A preliminary filtering (color filtering and binarization) was applied on images of Figure 3A, following the steps given in Figure 2A. In parallel, those example surfaces were topography-profiled (i.e., height profile) using VHX-1000 microscopy suite. They are named hereby as T-profiles (Figure 3B,C). T-profiles consisted of a combination of saturation-brightness-contrast revealing the surface texture [13]. They were used for qualitative discrimination of signals belonging to undesired patterns. Those undesired features were resin bubbles and holes, polishing scratches, bright spots of reflective material or polishing paste residues inside holes. They were also matched with their corresponding color profiles using Color Profiler [14] (Figure 3D) to discriminate accurately between char specimen and epoxy resin. T-profiles have been reconstructed by the microscopy suite, so, their accuracy was not discussed here (because the through-focus imaging and its subsequent aberrations are out of the scope of study [18]). Profile plots of Figure 3 contribute only to a global understanding of texture versus color signals and their intensities.

The color perception depends on the properties of the light source, of the illuminated material and of the propagation paths of the reflected light from the object. First, as mentioned earlier, the object was illuminated with a white light source, co-axial with the recording target. This enabled a rather uniform light intensity, thus a uniform recording of reflected light over the surface area. This is easily noticeable through RGB profiles, especially of Specimen #2, plotted in Figure 3D. Second, the reflectance properties of the object were modified by introducing the color pigments. The pigments provided a distinct red hue (also on HSB plots), as observed in the encircled regions **I** of profiles in Figure 3D. So, surface area representing voids could be easily distinguished from specimen cross-section. Third, the perception of light intensity depended on the propagation path: the pigments increased the resin opacity and limited the propagation of light inside the semi-transparent resin. This reduced multiple scattering in the resin embedded sections of chars, especially from the specular surfaces of char additives (e.g., graphite flakes). This, in turn, enhanced the contrast, even on less pigmented sections containing smallest micropores, as observed in the encircled regions **II** of profiles in Figure 3D. Finally, the very high intensity signals were thresholded because they represented the reflective materials in pores (either the diamond paste during surface polish or reflective char material, both susceptible to be present in non interconnected pores) as depicted in the encircled regions **III** of profiles in Figure 3D. Thresholding of the above aberrant signals leaded to the binarized images of Figure 3E. Their corresponding profile plots through the yellow lines are depicted in Figure 3F. Of course, there are some uncertainties due to non interconnected pores and high reflective materials inside the char (e.g., mineral additives of Specimen #1 and expanded graphite flakes of Specimen #2). Nevertheless, those did not affect the image analyses and will be shown in Section 3.4.

### 3.2. Optical Image Filtering

As shown in Figure 3, color retrieval alone is insufficient for an accurate extraction of resin embedded cross-section. This is due to many phenomena, including: local uneven illumination from reflective materials inside coating specimens, some disconnected pores filled with reflective substances due to material properties or due to polishing, some surface rugosity due to scratches during handling of specimen, smallest micropores where the pigments could not permeate. Those features are discriminated by looking at signal histogram peaks on digital microscope recordings. Pixel signals can be gathered under variable color systems. Non exhaustive examples are HSB (used here in image retrieval), RGB (given above in profile plots), L*a*b* (closest to human visual perception). RGB is a trichromatic description [19] (as in plots of Figure 3D): each pixel shall be identified according to its levels of red/green/blue and gathered under histograms before any image filtering. It is based on linear descriptors (addition, subtraction) of each channel, so, it is useful to generate colors but not to filter them. Indeed, colors shall be treated with their intensities for a complete identification (e.g., a dull red shall be treated differently than a bright red because this discrepancy hints at physical phenomena). HSB is a transformation system on a cylindrical space covering all possible ranges [19]. L*a*b* is an international standard adjusted to human eye gamuts, so that the color perception is device independent on displays and monitors [19]. Thereby, HSB was the most suitable in this study because low intensity colors can appear gray to human perception, whereas S (saturation) signal is capable of probing the difference [16].

Sequential masking of undesired features was performed as illustrated in Figure 2. Here, resin casted specimen #1 had an analyzed surface area of approximately 2 cm^2^ (Figure 2B). Resin casted specimen #2 (Figure 2C) had a larger analyzed surface area of approximately 4 cm^2^. A 3 × 3 median filter was applied to remove noise on histograms. The first filter consisted of color hue thresholding with high saturation, thanks to red pigmented resin. The second filter could be applied if necessary, depending on the resin and the specimen behaviors. For example it could be violet or orange hue, if the casting resin was not optically clear enough and had a bluish or yellowish color. Then, a S&B (saturation and brightness) filter was necessary to remove undesired features depending on the specimen porous structure. This will be detailed in Section 3.4. Finally, the color-thresholded image was transformed to grayscale and binarized, in order to obtain a BW (black and white) image for the fractal analyses below. As presented in the bottom images of Figure 2B,C (original images superposed with their mask), the char skeleton could reasonably be extracted by image retrieval technique.

### 3.3. Fractal Analyses and Comparison to CT Results

Intumescent specimens can be considered as porous media, but their physical properties (e.g., density, void ratio or porosity) given by CT or optical images are only apparent and limited by imaging resolution, and one shall use up/down-scaling and homogenization techniques. Therefore, another structural parameter is needed for quantitative comparison of optical technique with CT results. This parameter shall be scale invariant, because CT and optical recordings have different resolutions. Here, fractal dimension was a good candidate because it indicated both the mass density distribution and the morphology autocorrelation with scale invariance.

The power laws were determined following the correlation $D_{f}=log\left(N\right)/log\left(1/a\right)$, where *a* is the variable box dimension in pixels, N is the minimum number of boxes needed to encompass the whole object that contained the material. The box covering was performed on the surface area (i.e., not limited to pore boundaries representing the tortuosity), because we are interested in the mass density of material. $D_{f}$ with this method can be equal to a maximum of 2 in 2D-space. In practice, numerical computations shall be saturated at a slightly lower value [17], in our case the box counting saturated at $D_{f}=1.965$ for the slope of 2D-space filling curve.

**Figure 4:**
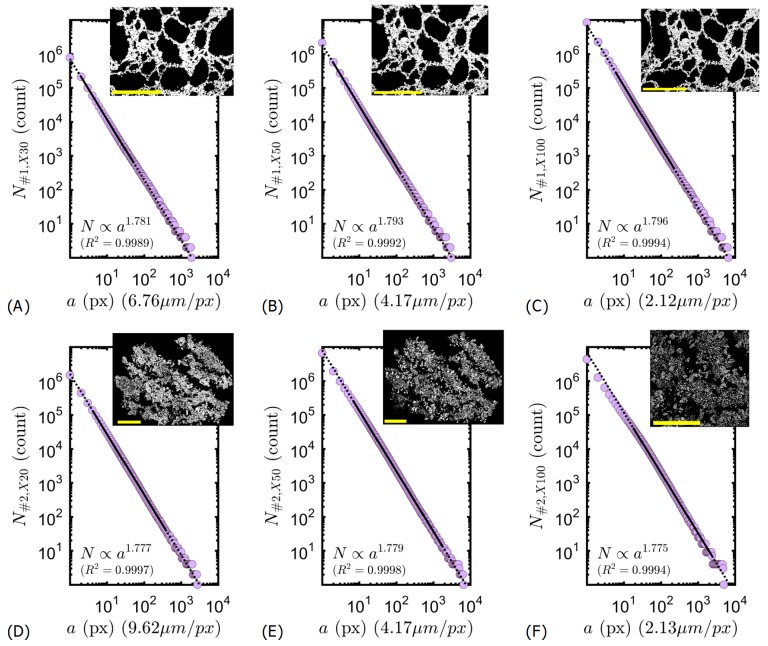
Box count results. Specimen #1: Epoxy char cross-section at (**A**) ×30, (**B**) ×50, (**C**) ×100 magnifications. Specimen #2: Silicone char cross-section at (**D**) ×20, (**E**) ×50, (**F**) ×100 magnifications. All scale bars are 5 mm.

Analyses were repeated on binarized images recorded at different magnifications. Results are presented in Figure 4. Linear regressions (indicated by solid lines) gave similar value of fractal dimension for the specimen at variable magnifications. Accordingly, the fractal dimension of Specimen #1 was determined as $D_{f,1}=1.788\pm 0.008$ as plotted in Figure 4A–C. The fractal dimension of Specimen #2 was determined as $D_{f,2}=1.777\pm 0.002$ as plotted in Figure 4D–F. As observed clearly in Figure 4F, the power law fails below few tens of μm; this lower limit of fractality complies with the lower limit determined by CT data [8]. Scale-invariance and cut-off are validated.

**Figure 5:**
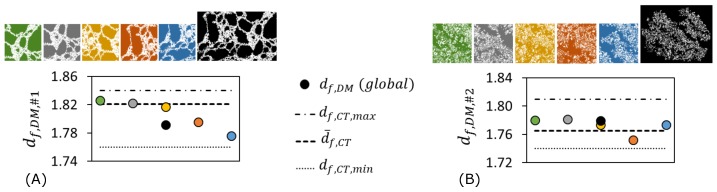
Self-similarity and comparison with CT, optical data at X50 magnification: (**A**) Specimen #1, (**B**) Specimen #2. Fractal dimension through the example cross-section is obtained from optical microscopy image: this shows simultaneously the self-similarity and the variation of fractality around a mean value for data obtained from optical digital microscopy. Tomography data is adapted from previously reported results by CT/HRCT [8] on same types of specimens. Subscripts: #1 is for epoxy-based specimen, #2 is for silicone-based specimen, CT is for computed tomography, DM is for optical/digital microscopy.

Also, the results at ×50 magnification were correlated with their CT/HRCT equivalent, reported in the same region of similar specimens [8]. Results are plotted in Figure 5A,B for Specimens #1 and #2 respectively. Here, different colors represented subsections near the same region. It is clearly seen that the self-similarity is more or less conserved for both specimens, and the optical results were within the maximum and minimum limits of $D_{f}$ determined by CT (regular and high resolution) within the same region on similar intumescent speciments [8]. It is reminded that the variation of CT computed $D_{f}$ was due to the slight multifractal behavior of intumescence; local values of $D_{f}$ shall fluctuate around a global value assigned to each specimen type [8]. The absolute value of fluctuation was shown to depend on the mode of expansion, on temperature gradients in the furnace and on boundary effects [8]. So, the differences observed in Figure 5 are due to slight inhomogeneity of the system in terms of time dependent materials properties, i.e., its rheological behavior. The volumetric fluctuations around the global values of $D_{f,1}$ and $D_{f,2}$ are quantified as: $\Delta D_{f,1,CT}\approx 0.08$ and $\Delta D_{f,1,optical}\approx 0.06$ for Specimen #1 (Figure 5A); $\Delta D_{f,2,CT}\approx 0.06$ and $\Delta D_{f,2,optical}\approx 0.03$ for Specimen #2 (Figure 5B). CT covered all the layers including crust, middle, active layer. Optical values are smaller because they report local volume in the middle layers. Unsurprisingly, $\Delta D_{f}$ is higher for Specimen #1 because its bubbling expansion presented a complex rheological behavior [20]. This behavior leaded to non-uniform bubble distributions [21] and resulted in a complex geometry with variable local densities in Specimen #1. The fluctuation of $\Delta D_{f}$ is smaller for Specimen #2 due to its physical mode of action, because the expansion of each layer ocurred in similar conditions with similar constraints. Expandable graphite platelets were distributed rather homogeneously, expansion of all graphite worms occurred at similar temperature [20], and the movement was less constrained because the silicone binder was malleable (contrary to previous formulations [20]). Finally, $D_{f}$ and $\Delta D_{f}$ values computed from the optical method comply with CT data. It demonstrates the effectiveness of the optical method.

### 3.4. Note on the Effect of Threshold Intensities

Contrary to digital microscopy, X-ray CT requires more rigorous acquisition conditions (e.g., proper source type and target, right acquisition time, precise stage holder) and reconstruction parameters (e.g., sufficient angular steps, adequate reconstruction and noise filtering algorithms). On CT reconstructed slice images, the grayscale threshold is crucial because it contains the only information about the existence or not of solid material in each pixel. In that sense, color contrasted imaging provided a less critical playground, because the information is projected to three intensity profiles instead of one (i.e., color hue, saturation and brightness).

**Figure 6:**
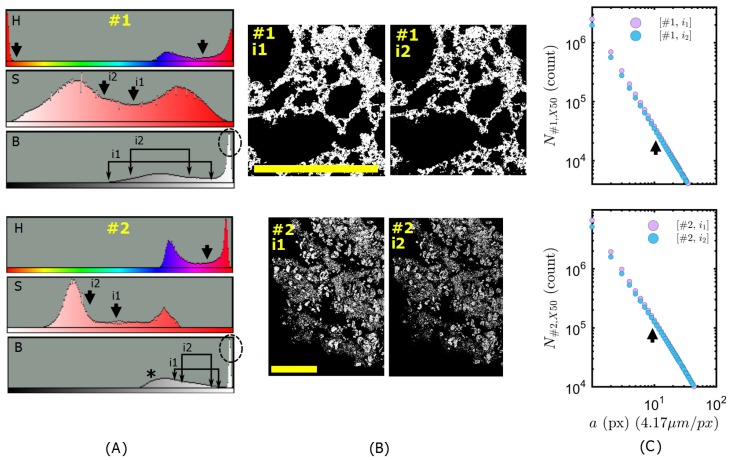
Tresholding histogram peaks at **i1** and **i2** levels of signal intensity, in order to retrieve specimen skeleton. (**A)** Examples of HSB histograms for specimen types #1 and #2. (**B**) Zoomed sections of thresholded binarized recordings. Scale bars represent 5 mm. (**C**) Zoom at box counts: Linear regression, i.e., fractal dimension, remain unchanged down to the resolution indicated by the arrow.

Nevertheless, the binarized skeleton of color contrasting was subject to slight uncertainties due to choice of pigments (size and color) and due to threshold intensities in HSB color space. Once the color contrast was ensured (arrows on H-histograms, Figure 6A), the separation between the “specimen” peak and the “pore” peak were subject to some uncertainty, as illustrated in the B- and S-histograms of Figure 6A. Minimum and maximum amounts of thresholding are defined by i1 and i2 levels respectively, as indicated in Figure 6A. For Specimen #1, the pigments impregnated almost all the voids because the pores were in bubbly form and highly interconnected. Indeed, the automated thresholding options (in ImageJ) for B histogram did not detect any background separation. Here, the problematic part was the intrinsic color of char specimen (grayish-yellowish) which shifted the color hue towards the red-yellow range in H histogram, so, approaching color saturation peaks (see S histogram of Specimen #1 in Figure 6A). For Specimen #2, the impregnation of pigments was prevented by the complex mixture of graphite platelets mixed with silicone binder and other additives. Therefore, it was not possible to mask the specimen skeleton only by color filtering. Indeed, the automated methods (ImageJ Iso Data [15]) detected a threshold level between the object and the background (see B-histogram of Specimen #2 in Figure 6A, background peak indicated by a star). This is due to non-pigmented sections, inside the resin, giving a darker brightness shade. Thanks to the increased opacity of casting resin with pigments, we were able to distinguish those features from surface/cross-section.

The corresponding image densities are observed on zoomed sections depicted in Figure 6B. In all cases, the solid skeleton was correctly defined, because the box count (i.e., the fractal dimension) remained unchanged down to the resolution indicated by the arrows in Figure 6C. This corresponds approximately to ≈ 54 μm for Specimen #1 and to ≈ 41 μm for Specimen #2. Values are in accordance with CT/HRCT indicating lower fractality limits between 32–64 μm [8]. This is not surprising, because the binarized structure is similar to that observed in Figure 6B. The reason is that the size of the uncertain details are smaller than the size of geometrical building blocks of skeleton; therefore, the quantitative analyses above remain unchanged, unless a correct computation of image computed void ratio would be needed.

On the other hand, the image computed void fraction Φ varied between $\Phi_{1}\left(i1\right)=0.72$ and $\Phi_{1}\left(i2\right)=0.77$ for different thresholds on Specimen #1 (against $\Phi_{1}\approx 0.4$ from CT [8]); between $\Phi_{2}\left(i1\right)=0.82$ and $\Phi_{2}\left(i2\right)=0.86$ for different thresholds on Specimen #2 (against $\Phi_{2}\approx 0.5$ from CT [8]). It was computed using the image computed probability of finding material based on pixel count. Please note that those values are much closer to the reported realistic values of ≈85–90% in the heating temperature range of 400–450 °C reported on similar intumescent coatings [22]. Indeed, the resolution of regular CT was 81 μm/pix (against 2 to 10 μm/pix in optical method), so, CT was not capable of probing the micro porosity even though the micro (fractal) tortuosity was reasonably detected. Thereby, in color contrasted images, the image computed void ratio is far more realistic than the literature values given by computed tomography [8,23]. So, the color contrast method could be improved in the future as a rapid complementary tool to the measurements of apparent density properties on fire tested residues.

## 4. Conclusions

Macroscale and mesoscale inner structures of intumescent fire protective coatings are interesting for fire safety research. Non-destructive techniques as computed tomography shall have some drawbacks due to loss of signals/information for some specimens and due to their cost for regular use. In this study, we proposed a cheaper and practical way of optical image retrieval using color contrast. Intumescent chars were casted into red pigmented epoxy resin, imaged under optical digital microscopy. The color contrasted images were treated using a sequential metrology to mask the char cross-section and perform quantitative analyses. Fractal analyses were performed on binarized optical images, giving a scale independent and quantitative morphology parameter. Optical results were compared to their equivalent power laws from X-ray CT. Results of the new metrology complied with previous scale-invariant parameters. Therefore, the structural integrity of brittle specimens was shown to be successfully maintained in resin casting. This proposed method provides a facile tool to analyze the cross sections of our carbonaceous porous residues with minimized perturbation on structural data.

This technique can be used for preliminary analyses of large sets of fire tested specimens with low cost. It is also complementary to other experimental techniques examining the structural data such as rheology and porosimetry. Please note that this imaging technique can provide more realistic/reliable physical data, e.g., density and tortuosity, compared to X-ray CT, due to the flexibility of optical technique in terms of resolution and of specimen size. Results also were more realistic than CT compared to reported experimental data of pore fraction. In the future, it can be used for porosity/tortuosity analyses down to the resolution limit of the optical digital microscopy, with proper selection of coloring/pigments, of appropriate surface treatments, and of adapted magnifications/filtering during imaging. Method can also be applied to other types of macro- and microporous materials and polymers in parallel with engineering standards.
